# Identification of ubiquitination-related hub genes in chronic myeloid leukemia cell by bioinformatics analysis

**DOI:** 10.7150/jca.96405

**Published:** 2024-05-20

**Authors:** Qian Zhou, Zhuoran Li, Li Meng, Ying Wang, Muhammad Sameer Ashaq, Yuan Li, Baobing Zhao

**Affiliations:** 1 Key Lab of Chemical Biology (MOE), School of Pharmaceutical Sciences, Cheeloo College of Medicine, Shandong University, Jinan, Shandong, 250012, China.; 2 NMPA Key Laboratory for Technology Research and Evaluation of Drug Products, School of Pharmaceutical Sciences, Cheeloo College of Medicine, Shandong University, Jinan, Shandong, 250012, China.; 3 Department of Pharmacology, School of Pharmaceutical Sciences, Cheeloo College of Medicine, Shandong University, Jinan, Shandong, 250012, China.

**Keywords:** Chronic myeloid leukemia stem cell, Ubiquitination, FANCD2, UHRF1, CDC20

## Abstract

**Purpose**: Chronic myeloid leukemia stem cells (CML-LSCs) are posited as the primary instigators of resistance to tyrosine kinase inhibitors (TKIs) and recurrence of CML. Ubiquitination, a post-translational modification, has been implicated in the worsening process of CML. A more detailed understanding of their crosstalk needs further investigation. Our research aims to explore the potential ubiquitination-related genes in CML-LSC using bioinformatics analysis that might be the target for the eradication of LSCs.

**Methods**: The ubiquitination modification-related differentially expressed genes (UUC-DEGs) between normal hematopoietic stem cells (HSCs) and LSCs were obtained from GSE47927 and iUUCD database. Subsequently, the hub UUC-DEGs were identified through protein-protein interaction (PPI) network analysis utilizing the STRING database and the MCODE plug-in within the Cytoscape platform. The upstream regulation network of the hub UUC-DEGs was studied by hTFtarget, PROMO, miRDB and miRWalk databases respectively. Then the correlation between the hub UUC-DEGs and the immune cells was analyzed by the CIBERSORT algorithm and "ggcorrplot" package. Finally, we validated the function of hub UUC-DEGs in CML animal models, CML cell lines and CD34^+^ cells of the GSE24739 dataset.

**Results**: There is a strong association between the 4 hub UUC genes (*AURKA, Fancd2, Cdc20* and *Uhrf1*) of LSCs and the infiltration of CD4^+^/CD8^+^ T cells, NK cells and monocytes. 8 TFs and 23 miRNAs potentially targeted these 4 hub genes were constructed. Among these hub genes, *Fancd2, Cdc20* and *Uhrf1* were found to be highly expressed in CML-LSC, which knocking down resulted in significant inhibition of CML cell proliferation.

**Conclusions**: From the perspective of bioinformatics analysis,* UHRF1* and *CDC20* were identified as the novel key ubiquitination-related genes in CML-LSCs and the pathogenesis of CML.

## Introduction

Chronic myelocytic leukemia (CML) is a clonal myeloproliferative disorder of hematopoietic stem cell (HSC), which is mainly driven by the fusion gene BCR-ABL1, a product of the Philadelphia chromosome (Ph) 1. CML accounts for 0.3% of all cancers and 15% of adult leukemia, with a global incidence of 1.6-2 per 100,000 2. The abnormal tyrosine kinase activity of BCR-ABL1 leads to autophosphorylation and the phosphorylation of its substrate proteins, which activates the signaling pathways required for clonal proliferation of CML cells, leading to cell carcinogenesis 3. Although TKIs that target BCR-ABL1 kinase can alleviate symptoms, acquired resistance and residual leukemia stem cells (LSCs) remain clinical challenges for CML therapy 4.

Multiple post-translational modifications have been identified for BCR-ABL1 and its downstream signaling proteins, which play roles in the progression of CML, including phosphorylation, acetylation, methylation and ubiquitination 5-11. The ubiquitin-proteasome system is an important regulatory pathway for the intracellular protein degradation. Polyubiquitination and proteasome degradation of substrate proteins affect or regulate a variety of cellular activities, including transcription, inflammatory and immune response 12-15. Protein ubiquitination is a dynamic bidirectional process regulated by ubiquitination and deubiquitylation. Some ubiquitination-related proteins have been reported as potential drug targets for leukemia 16, 17 for their functional roles in the progression of leukemia.

A potential approach to address these challenges involves targeting key proteins involved in BCR-ABL1-driven signaling pathways through ubiquitination and deubiquitylation regulation, offering a promising strategy for CML treatment. Indeed, a previous study reported that BCR-ABL1 degradation could overcome TKIs resistance, which is achieved by targeted protein degradation mediated by targeted proteolytic chimeras (PROTAC) 18. This study found that the expression of several ubiquitination-related genes increased in CML, which may facilitate the discovery of therapeutic targets for CML.

## Material and methods

### Data source

**Figure 1:**
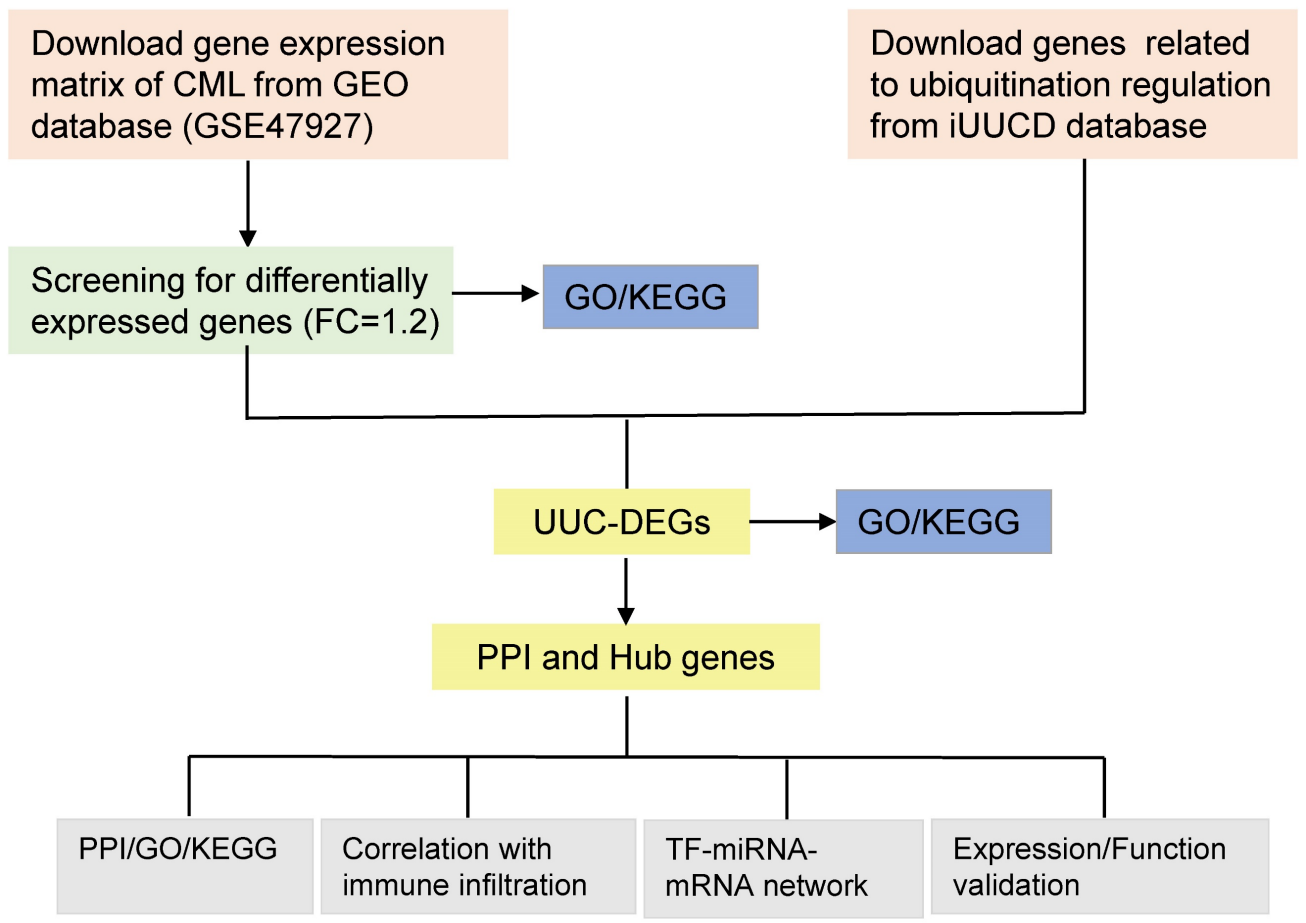
** Flow chart of this study.** FC, Fold Change; DEGs, differentially expressed genes; PPI, Protein-protein interaction; UUC, Ubiquitin and Ubiquitin-like Conjugation; GO, Gene Ontology; KEGG, Kyoto Encyclopedia of Genes and Genomes; TF, Transcriptional factors; miRNAs, microRNAs.

The gene expression profile data (GSE47927) were obtained from the Gene Expression Omnibus (GEO) database. 3 normal patient samples were compared with 12 CML samples including 6 CP, 4 AP and 2 BC samples. The data were visualized by the Origin2023b database. DEGs data was filtered by NetworkAnalyst (FC > 1.2, P< 0.05). The volcano map expressing the data was drawn by GraphPad Prism 8 and the top 40 genes were drawn in a heat map by the Morpheus website. According to Table S1, ubiquitination modification-related genes were obtained from iUUCD. It contained 1597 genes associated with ubiquitination modification, which included 9 E1s, 43 E2s, 919 E3s, 126 DUBs, 387 UBD and 113 ULDs. However, we only selected 1387 genes after filtering the duplicate values (Figure 1).

### Functional enrichment analysis

To explore the biological process and potential signaling pathways of DEGs in our model, GO and KEGG analyses were performed by NetworkAnalyst and Metascape.

### Identification of ubiquitination modification‑related DEGs (UUC-DEGs)

Ubiquitination modification-related genes (Ubiquitin and Ubiquitin-like Conjugation) were obtained from iUUCD which contained 1387 genes associated with ubiquitin. UUC-DEGs were identified by intersecting the DEGs from GSE47927 and the 1387 genes related to Ubiquitin and Ubiquitin-like Conjugation using a Venn Diagram.

### Protein-protein interaction network (PPI) analysis and hub genes obtain

To make a further understanding of the regulatory network of ubiquitination modification in CML, the overlapped UUC-DEGs were processed for PPI analysis with the STRING database which was reported to have the greatest ability to recover a diverse collection of disease-associated gene sets among 21 human gene-gene interaction network databases and the resulting interactions were visualized as a network using Cytoscape 3.9.1. The hub UUC-DEGs were identified using the MCODE plug-ins within Cytoscape 3.9.1.

### Immune infiltration analysis

The hub UUC-DEGs matrix was made the further immune infiltration analysis by the CIBERSORTx online database which included the expression matrix of 22 immune cells in the annotation file LM22. Differential analysis was conducted to investigate the relationship between DEGs and immune cells, as well as to determine the correlation between the Hub UUC-DEGs and immune cells, to calculate the correlation between the Hub UUC-DEGs and the immune cells, the CIBERSORT results file obtained by CIBERSORT algorithm and the Hub UUC-DEGs file are simultaneously executed by the correlation coefficient matrix “corr_mat <- cor (data,cibersort_result,method= "spearman")”, finally, plot the correlation matrix with the "ggcorrplot" package in R software.

### Hub UUC-DEGs‑TF‑miRNAs network prediction

To explore the upstream regulators of the hub UUC-DEGs, TFs of hub UUC-DEGs were predicted with the hTFtarget and PROMO database. miRNAs of the hub UUC-DEGs were predicted using the miRWalk and miRDB database. The TFs and miRNAs from the two databases were then intersected respectively. Finally, the network of hub UUC-DEGs, transcription factors and miRNAs were displayed via Cytoscape3.9.1.

### Cell culture

K562 cells stored by our laboratory, and cultured in RPMI 1640 medium (BasalMedia, Cat #L210KJ) with 10% FBS (SeraPure, Cat #SE141-500). KBM5 cells were kindly provided by Jingxuan Pan (Sun Yat-sen University), and cultured in IMDM (Thermo, Cat #12440053) supplemented with 10% FBS.

### Plasmids Construction and Cell Transduction

Cloning of shRNA into the PLKO.1 retroviral vector was constructed as previously described 19. The virus was packaged using a 2nd generation packaging system in 293T, then the K562 and KBM5 cells were infected. Finally, cell proliferation was measured using Flow Cytometry (Agilent).

### RNA extraction & real time‑PCR

Total RNA was extracted from CML model tissue or CML cells by Trizol (Thermo,15596026) and reversely transcribed into cDNA using a reverse transcription kit (Vazyme #R323). Real time‑PCR was performed with a SYBR Green (Vazyme #Q711) and the primers were shown in Table S2. The expression of the target gene relative to the reference gene is 2^ΔΔCt^.

### CML model

For the CML model, c-Kit^+^ BM cells from 8 weeks C57 mice were transduced with the MSCV-BCR-ABL1-IRES-EGFP and MSCV-IRES-EGFP vector retrovirus two rounds, followed by tail vein injection into irradiated (5.5Gy) recipient mice. The Lineage^-^ cells of the CML model and control group mice bone marrow were purified at 2-3 weeks after bone marrow transplantation.

### Statistical analysis

All data were shown as mean ± standard deviation, and analyzed using Prism 8.0 (GraphPad). The statistical significance of differences in groups was performed with Student's t-test and Two-way ANOVA. P < 0.05 was considered to be statistically significant.

## Results

### DEGs identified in BM-derived normal HSC and CML-LSC

The flowchart of this study is shown in Figure 1. To identify ubiquitination modification-related hub genes in CML-LSC, we first identified the differentially expressed genes (DEGs) with the GSE47927 datasets and performed GO and KEGG analysis. DEGs were then intersected with the ubiquitin and ubiquitin-like conjugation (UUC)-related genes obtained from the iUUCD database. Hub UUC-DEGs were identified via PPI network analysis, followed by the GO/KEGG enrichment analysis, correlation with immune infiltration analysis and TF-miRNA-mRNA network analysis. Finally, the expression and function of these hub UUC-DEGs were verified in CML animal models and cell lines.

**Figure 2:**
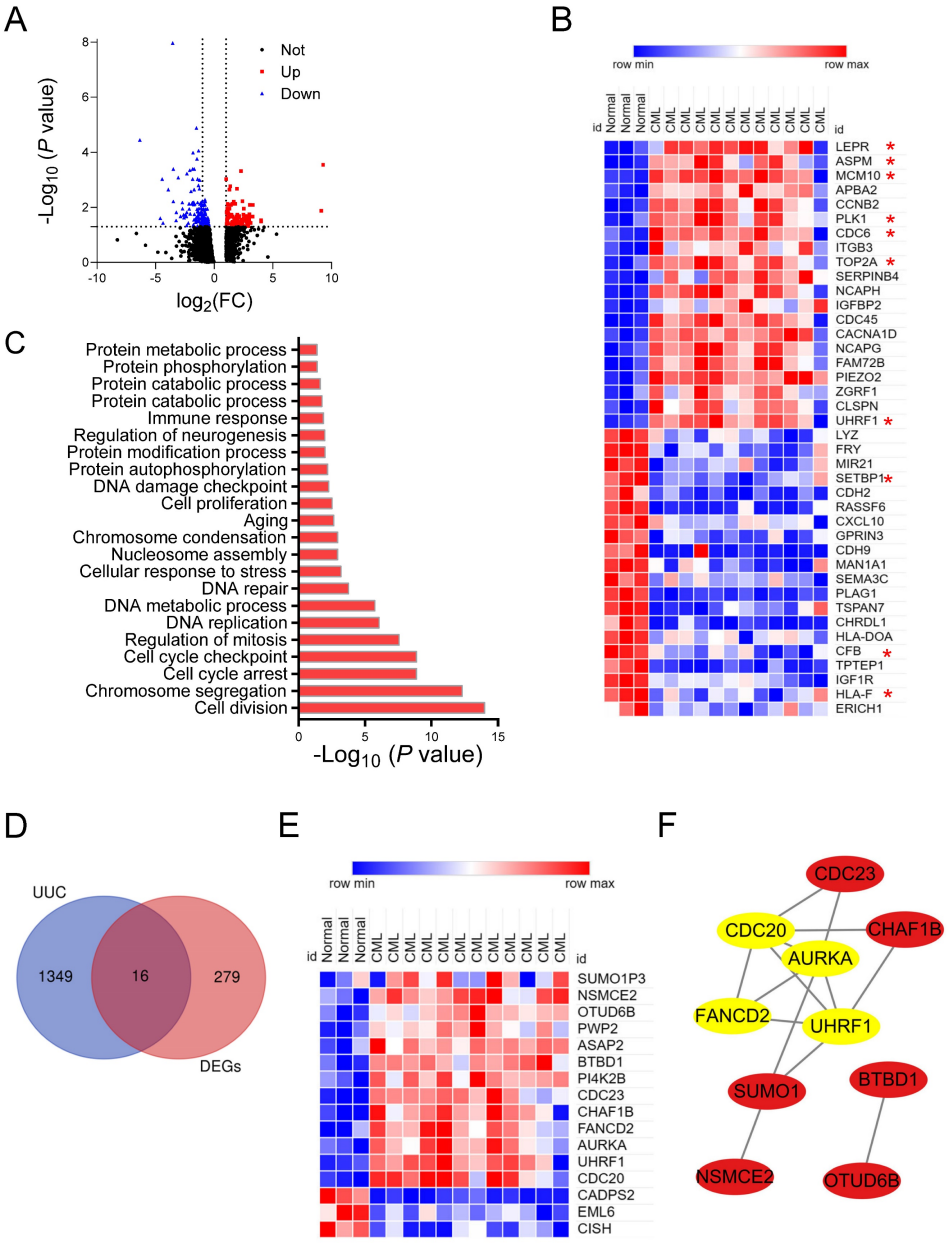
** Identification of UUC-DEGs and hub UUC-DEGs in CML.** (A) The volcano plots of DEGs in GSE47927. Red dots represent up-regulated genes, blue dots represent down-regulated genes and black dots represent not significant genes. (B) Heat map for top 40 DEGs in normal and CML samples. (C) The GO enrichment analysis of DEGs in GSE47927 by the NetworkAnalyst database. (D) The Venn diagram of gene expression profile data and DEGs in GSE47927 and UUC-related genes downloaded from iUUCD database. (E) Heat map for UUC-DEGs in normal and CML samples. (F) Construction of a PPI network of UUC-DEGs using Cytoscape. The top 4 hub UUC-DEGs were identified by the MCODE plug-in of Cytoscape software and marked with yellow.

The expression profiling of 3 BM-derived normal HSC and 12 CML-LSC was analyzed from the GSE47927, which included 150 upregulated and 145 downregulated genes (Figure 2A and Table S3). Moreover, top 40 DEGs were shown in Figure 2B.

### Functional analysis of DEGs

DEGs were then processed for the GO and KEGG analyses by the Networkanalyzd database. The most enriched biological processes were the cell cycle, DNA replication/damage/repair, and protein catabolic process (Figure 2C). KEGG enrichment analysis showed that these DEGs were mainly involved in the cell cycle, cell senescence, p53 pathway, Hippo pathway and FoxO pathway (Figure S1A). These results indicated that CML-LSC had distinct characteristics compared with normal HSCs, including active cell cycle, protein metabolism and activation of corresponding signaling. This is in line with the strong division and proliferation ability of CML cells and their obstruction of cell differentiation and maturation.

### UUC-DEGs identification in CML-LSC

According to above analysis results, protein metabolism process was significantly altered during the transformation from normal HSC to CML-LSC. Ubiquitination modification is an important way to regulate the stability, localization and function of proteins, thus affecting the physiological process of CML cells 18, 20. We intersected DEGs with UUC-related genes, and identified 16 overlapping genes (UUC-DEGs) (Figure 2D-E, Table S4). PCA results show that these UUC-DEGs own good reproducibility in normal HSC to CML-LSC (Figure S1B-C). Enrichment analysis demonstrated that these UUC-DEGs were mainly involved in the protein ubiquitination/sumoylation, DNA repair, and cell cycle (Figure S1D). KEGG enrichment also showed the enriched cell cycle, post-translational protein modification and metabolism of proteins (Figure S1E). Furthermore, based on the PPI network results of 16 UUC-DEGs, 4 hub genes were confirmed by the MCODE plug-in of Cytoscape, including FANCD2, AURKA, CDC20 and UHRF1 (Figure 2F).

### Immune cell infiltration in CML

**Figure 3:**
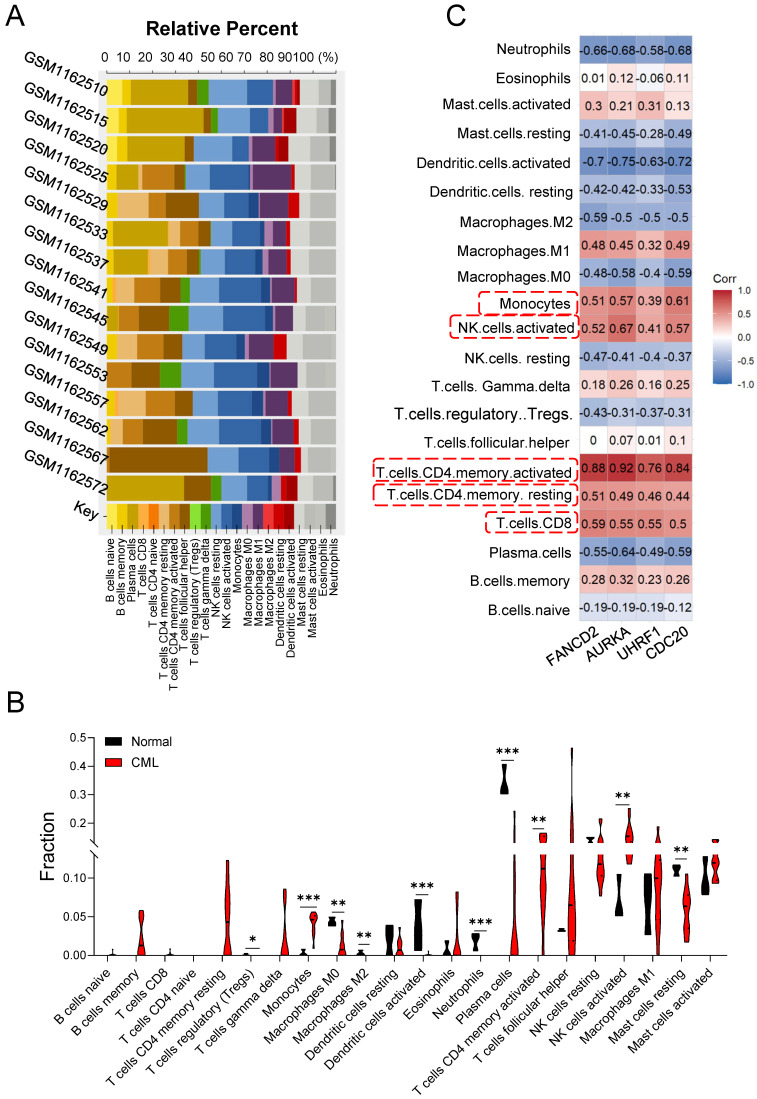

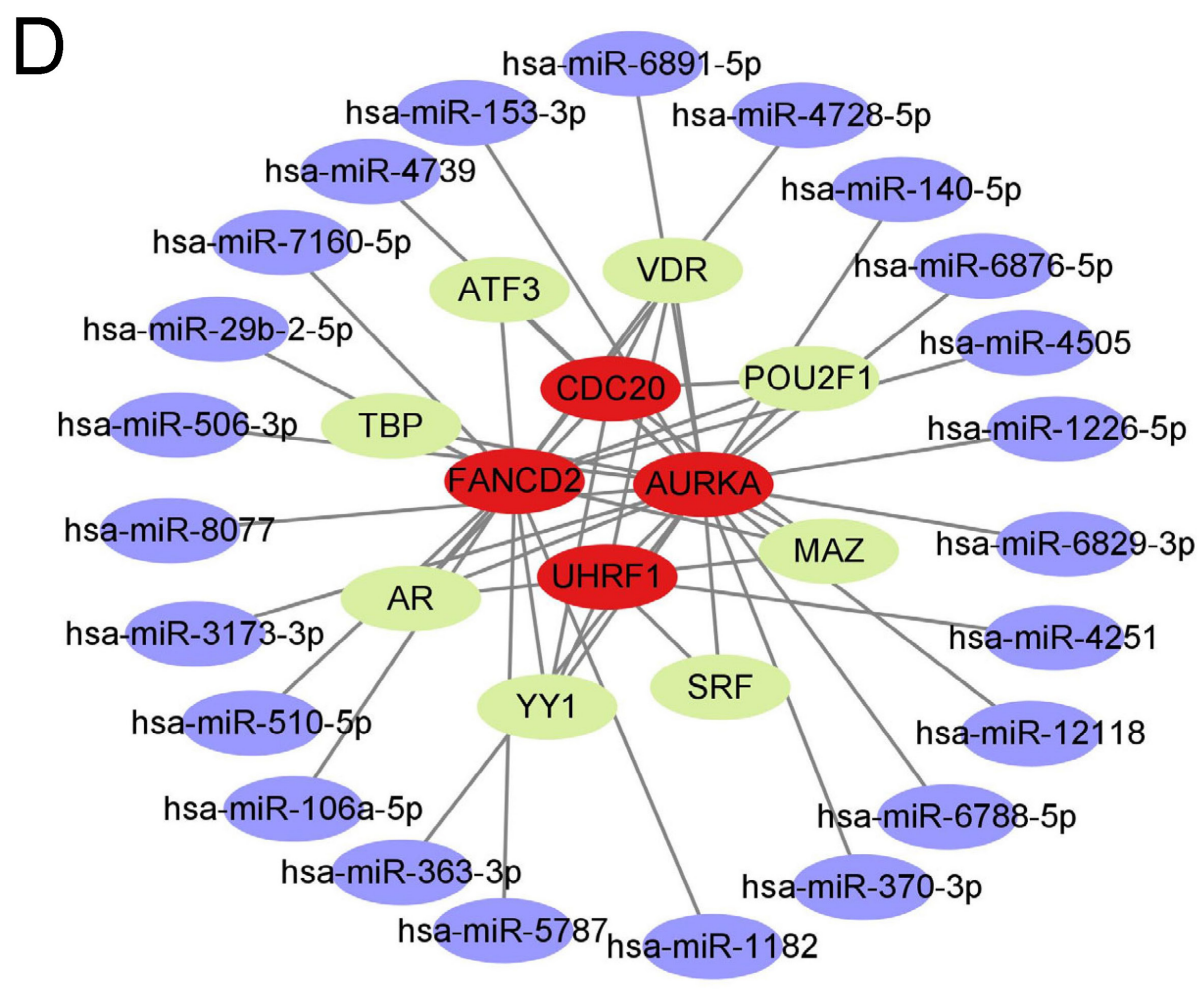
** Immune infiltration of the samples and TF-mRNA-miRNA network of hub UUC-DEGs.** (A) Stacked bar chart of the immune cell. (B) Violin diagram of the relative fraction of 22 immune cell types between two groups. *p < 0.05, **p < 0.01, ***p < 0.005. (C) The relationship between hub UUC-DEGs and immune cells. (D) TFs-mRNA-miRNA regulatory networks were constructed for CML based on the above analysis and their inside interaction relationships. The red color represents the mRNAs of hub UUC-DEGs. The purple color represents the miRNAs. The green color represents the TFs.

Bone marrow microenvironment (BMM) provides sanctuary for LSCs and protects them from the cytotoxic effects of chemotherapy 21, 22, which play important roles in CML cell growth, survival, proliferation, hemostasis, invasion and metastasis 23. To explore the difference in immune infiltration between normal hematopoiesis and CML LSCs, we used CIBERSORTx online tools to examine the correlation of immune cells with multiple GSM databases (Figure 3A). Compared with the normal group, the CML group showed a higher infiltration of the activated CD4^+^ memory T cells, activated NK cells, monocytes, accompanied by the low infiltration of Plasma cells, resting Mast cells, T cells regulatory, Macrophages M0, Macrophages M2, Dendritic cells and Neutrophils (Figure 3B). Furthermore, 4 hub genes expression were highly correlated with the infiltration of CD4^+^/CD8^+^ T cells, NK cells and monocytes (Figure 3C).

### Hub UUC-DEGs‑TFs‑miRNAs network

The upstream regulation of the hub UUC-DEGs was investigated by anticipating their transcriptional factors (TFs) and relative miRNAs. Two different databases were used and the intersection of both were identified as the potential TFs of hub UUC-DEGs (Table S5). MiRNAs of hub UUC-DEGs were also predicted with the miRWalk 3.0 and miRDB database. Together, the hub UUC-DEGs-TFs-miRNAs regulatory network was comprised of 8 TFs and 23 miRNAs (Figure 3D and Table S5).

### Validation of hub genes associated with ubiquitination in CML-LSC

**Figure 4:**
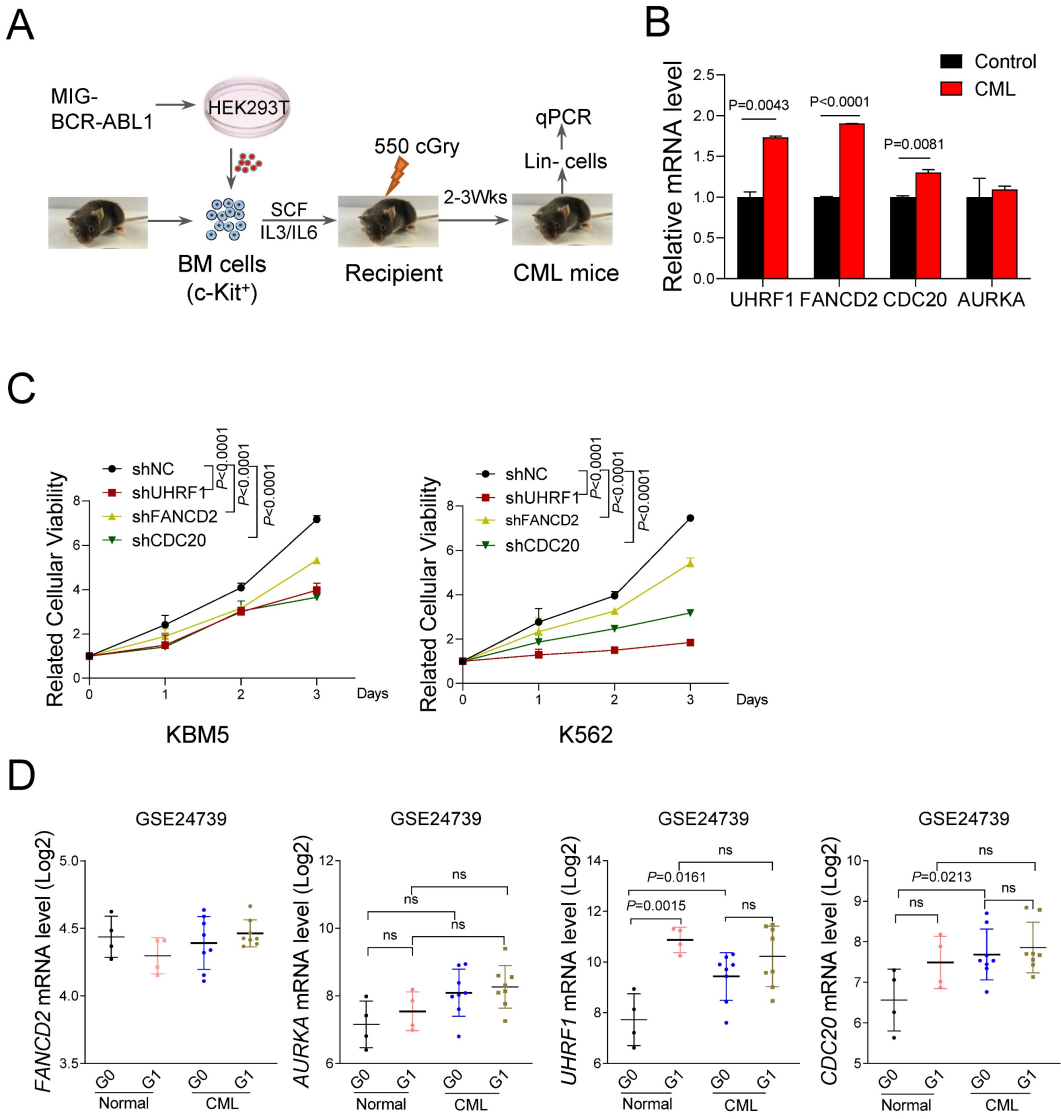
** Validation of hub UUC-DEGs in CML.** (A) Schematic strategy for CML mouse model. (B) Quantitative PCR analysis of hub UUC-DEGs mRNA level in lineage^-^ cells from control and CML mice as in A. Data were presented as mean±SD from three independent experiments. (C) Statistical analysis of cell proliferation in K562 and KBM5 cells with indicated shRNAs of hub UUC-DEGs. shNC represents a non-targeting shRNA. Data were obtained from three independent experiments. (D) The mRNA levels of hub UUC-DEGs in CD34^+^ cells based on GSE24739 analysis.

To further verify the expression of the 4 hub UUC-DEGs, we constructed a BM transplantation model with BCR-ABL1 ectopic expression (Figure 4A). Compared with the wild-type controls, CML mice showed a marked increase of *Fancd2, Cdc20* and *Uhrf1* mRNA level in BM Lineage^-^ cells but not *Aurka* (Figure 4B). To explore the functional roles of 3 hub UUC-DEGs in the pathogenesis of CML, we carry out the proliferation assay in CML cell lines transduced with the indicated shRNA. Compared with the control group, 3 hub UUC-DEGs silencing dramatically suppressed the proliferation of CML cell lines, respectively (Figure 4C).

In addition, we also examined the expression of hub UUC-DEGs using another dataset (GSE24739), in which HSCs were dissected into quiescent (G0 phase) and active (G1 phase) subpopulations. For the normal HSC, only *UHRF1* showed significant upregulation in G_1_-HSC compared with that of G_0_-HSC. Interestingly, both *UHRF1* and *CDC20* were highly expressed in the G_0_-LSC, even though they showed comparable expression in G_0_-LSC and G_1_-LSC (Figure 4D). Although *FANCD2* expression showed mild changes in the subpopulations of HSC, it had been documented as the upregulation of *FANCD2* in CML-LSC.

## Discussion

In our study, we first established a link between ubiquitination modification and CML-LSCs based on multiple ubiquitination modification-related genes, in which *UHRF1* and *CDC20* were identified as the novel potential target genes as well as FANCD2 previously well investigated in CML. Our study further supported that ubiquitination modification plays an important role in the progression of CML.

CML is caused by the self-renewal and rapid proliferation of CML-LSC and the block of myeloid cell differentiation 24, which was verified by the GO/KEGG enrichment results of CML-LSC, which showed the biological processes related to cell cycle, cell proliferation, DNA replication/repair and protein modification process. CDC20 has been reported to form E3 ubiquitin ligase APC^CDC20^ with Anaphase Promoting Complex (APC) to regulate cell cycle progression 25, 26. It has been reported that CDC20, as a substrate of Cdh1, was involved in the process of genomic instability by controlling the cell cycle of the G0/G1 phase 26. Based on previous studies, we speculate that CDC20 may be involved in the transition process of CML-LSCs from resting state to active state, which is worthy of further study.

Reactive oxygen species (ROS) and DNA damage increase during self-renewal and proliferation of CML-LSC. A previous study shows that monoubiquitinated FANCD2 facilitates the repair of numerous ROS-induced DNA double-strand breaks (DSBs) 27, and downregulation of FANCD2 delayed BCR-ABL1-derived CML in mice 27-29. Consistent with this study, we also found FANCD2 increased in CML mice and promoted the proliferation of CML cell lines. The conserved function of FANCD2 within the progress of CML was verified by our analysis and these confirmatory experiments.

The main reason that current strategies could not effectively eliminate LSCs lies in the frequently quiescent state of LSCs 24. UHRF1 is a ubiquitin-like domain protein encoded by a member of the UPR signaling pathway-related gene, and the protein is required for myeloid leukemogenesis by maintaining self-renewal of LSCs in AML 30 and ALL 31. It has been reported that UHRF1 was suppressed by dasatinib in K562 cells 32, which is consistent with our analysis results, in which UHRF1 increased in CML-LSC. These results suggest that UHRF1, as a key target gene, regulates the self-renewal of CML-LSC. It is an effective way to inhibit the expansion of LSCs by targeting UHRF1 and its function and related regulatory mechanisms are worthy of further study.

Innate immune response has been proven to be activated in murine CML and further enhanced by TKI or alpha-interferon treatment 33, 34. Bioinformatic studies in several cancers have shown that ubiquitination is implicated in immune infiltrates and pathways 35-37. In our study, CML is correlated with an impaired immune response, indicated by the low scores of T cells regulatory, Macrophages M2, activated dendritic cells, neutrophils, plasma cells and resting mast cells and the high scores of monocytes, activated CD4^+^ memory T cells and activated NK cells. Given that these 4 hub genes expression was highly correlated with the infiltration of CD4^+^/CD8^+^ T cells, NK cells and monocytes, it is rational that immune disorders partially contribute to the poor prognosis of CML patients.

Although we used many bioinformatics analyses to explore the relevance of ubiquitination to the progression of CML, there were still some limitations. Firstly, the samples were not sufficiently large due to the less related data in the GEO databases. Secondly, we need further functional research on the molecular mechanisms of these hub genes we identified in CML.

## Conclusion

In our results, we determined 4 hub genes closely associated with ubiquitination modification in CML. These findings would significantly contribute to the understanding of the crosstalk between ubiquitination modification and CML, offering promising avenues for investigating therapeutic interventions targeting ubiquitination-related proteins in CML.

## Supplementary Material

Supplementary figure, tables 2 and 5.

Supplementary tables 1, 3, 4.
