# Supplementary material for: Identification of ubiquitination-related hub genes in chronic myeloid leukemia cell by bioinformatics analysis
Source: J Cancer. 2024 May 20;15(12):3750–9. doi: 10.7150/jca.96405 (PMC11190763; doi:10.7150/jca.96405)
Supplement: Supplementary file 1 — Supplementary figure, tables 2 and 5. [file jcav15p3750s1.pdf]

Identification of ubiquitination-related hub genes in chronic myeloid leukemia cell by bioinformatics analysis

Qian Zhou[1,2,3], Zhuoran Li[1,2,3], Li Meng[1,2,3], Ying Wang[1,2,3], Muhammad Sameer Ashaq[1,2,3], Yuan Li[1,2,3], Baobing Zhao[1,2,3]#

[1]Key Lab of Chemical Biology (MOE), School of Pharmaceutical Sciences, Cheeloo College of Medicine, Shandong University, Jinan, Shandong, 250012, China;

[2]NMPA Key Laboratory for Technology Research and Evaluation of Drug Products, School of Pharmaceutical Sciences, Cheeloo College of Medicine, Shandong University, Jinan, Shandong, 250012, China;

[3]Department of Pharmacology, School of Pharmaceutical Sciences, Cheeloo College of Medicine, Shandong University, Jinan, Shandong, 250012, China

#Correspondence to:

Baobing Zhao, Ph.D., Department of Pharmacology, School of Pharmaceutical Sciences, Shandong University, NO. 44 Wenhua Road, Jinan, Shandong, P.R.China, 250012; baobingzh@sdu.edu.cn; TEL/FAX: +86-531-88382176.

Supplementary figure 1

(A) The KEGG enrichment analysis of DEGs in GSE47927 NetworkAnalyst.

(B) The scree plots of DEGs in GSE47927.

(C) The PCA of normal and CML samples.

(D-E) The GO and KEGG enrichment pathway analysis of UUC-related DEGs using the DAVID database.

Supp Fig1

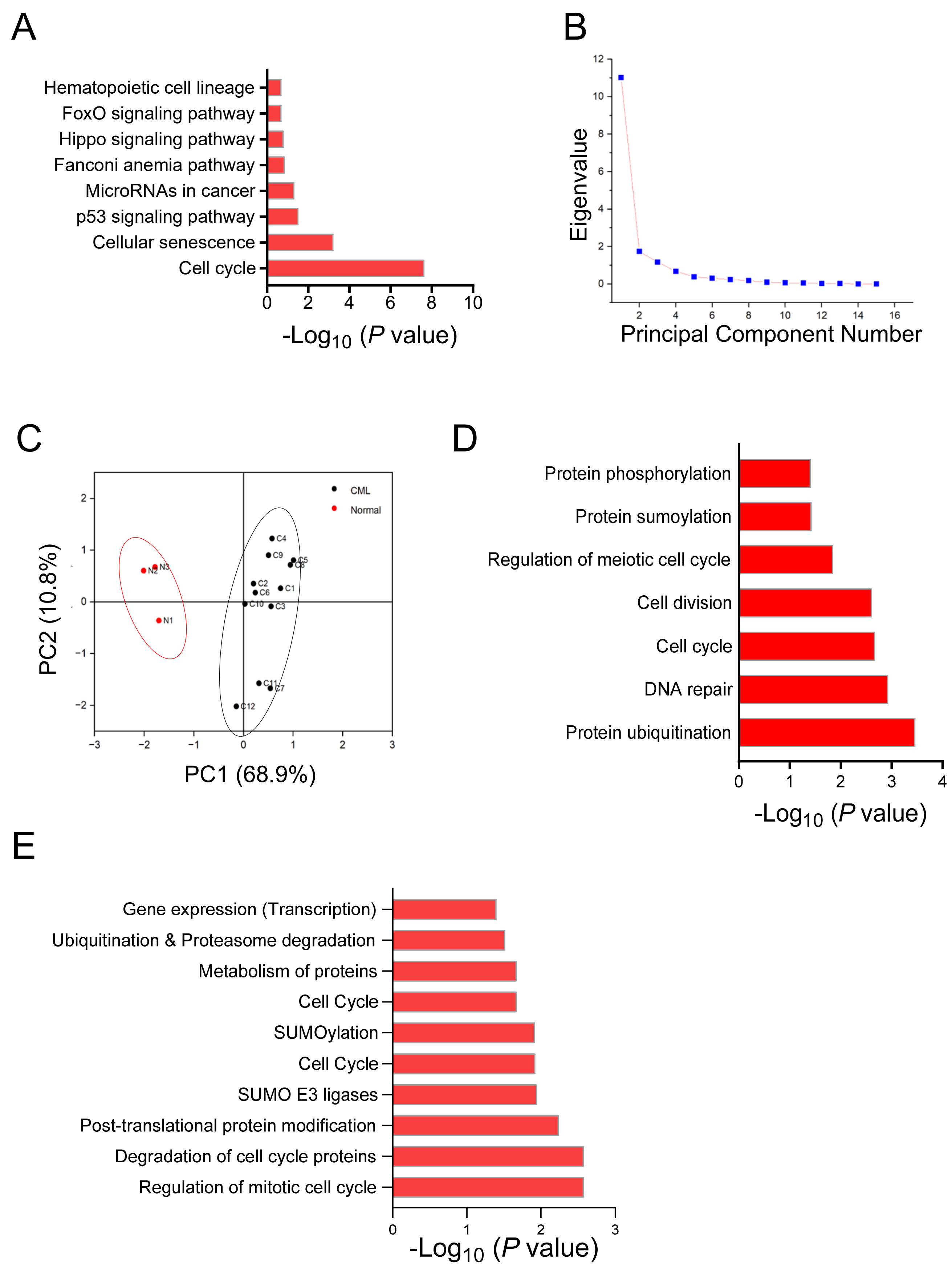
A
Hematopoietic cell lineage
FoxO signaling pathway
Hippo signaling pathway
Fanconi anemia pathway
MicroRNAs in cancer
p53 signaling pathway
Cellular senescence
Cell cycle
0 2 4 6 8 10
-Log10 (P value)
B
Eigenvalue
12 10 8 6 4 2 0
2 4 6 8 10 12 14 16
Principal Component Number
C
CML
Normal
N1 N2 N3
C1 C2 C3 C4 C5 C6 C7 C8 C9 C10 C11 C12
PC2 (10.8%)
PC1 (68.9%)
D
Protein phosphorylation
Protein sumoylation
Regulation of meiotic cell cycle
Cell division
Cell cycle
DNA repair
Protein ubiquitination
0 1 2 3 4
-Log10 (P value)
E
Gene expression (Transcription)
Ubiquitination & Proteasome degradation
Metabolism of proteins
Cell Cycle
SUMOylation
Cell Cycle
SUMO E3 ligases
Post-translational protein modification
Degradation of cell cycle proteins
Regulation of mitotic cell cycle
0 1 2 3
-Log10 (P value)

Table S2：Primers

Gene	Species	Sequence
FANCD2-F	mouse	CAAAATCAGCTAGGTGTGGATCA
FANCD2-R	mouse	CCAGGCCATTAACAAACTCTTCT
UHRF1-F	mouse	CCACACCGTGAACTCTCTGTC
UHRF1-R	mouse	GGCGCACATCATAATCGAAGA
CDC20-F	mouse	TTCGTGTTCGAGAGCGATTTG
CDC20-R	mouse	ACCTTGGAACTAGATTTGCCAG
AURKA-F	mouse	CTGGATGCTGCAAACGGATAG
AURKA-R	mouse	CGAAGGGAACAGTGGTCTTAACA
18S-F	Mouse/ human	GGAATATGCACCACTTGGAACA
18S-R	mouse/human	TAAGACAGGGCATTTGCCAAT
FANCD2-shRNA	human	CGTCTATTAGATTGGAGGATT
UHRF1-shRNA	human	ATGTGGGATGAGACGGAATTG
CDC20-shRNA	human	AGACCAACCCATCACCTCAGT

Table S5： Hub-UUB-DEGs/TFs/ miRNA network genes

**Hub-UUB-DEGs**	FANCD2； AURKA； UHRF1； CDC20
**TFs**	VDR； YY1； POU2F1； TBP；
	MAZ； SRF； AR； ATF3
**miRNA**	hsa-miR-106a-5p； hsa-miR-1182； hsa-miR-12118； hsa-miR-363-3p； hsa-miR-370-3p； hsa-miR-4251； hsa-miR-6788-5p； hsa-miR-6829-3p； hsa-miR-6876-5p； hsa-miR-140-5p； hsa-miR-153-3p； hsa-miR-29b-2-5p； hsa-miR-4728-5p； hsa-miR-4739； hsa-miR-506-3p； hsa-miR-4728-6p； hsa-miR-8077； hsa-miR-5787； hsa-miR-1226-5p； hsa-miR-510-5p； hsa-miR-3173-3p； hsa-miR-6891-5p； hsa-miR-4505
